# Circ_0099630 knockdown alleviates lipopolysaccharide-induced injuries of human periodontal ligament cells through the inhibition of TLR4 by releasing miR-409-3p

**DOI:** 10.1186/s12903-023-03622-7

**Published:** 2023-11-25

**Authors:** Hongyan Qi, Bing Han, Jin Che

**Affiliations:** 1 Department of Stomatology, First Hospital Affiliated to Lanzhou University, No.1 Donggangxi Rd, Chengguan District, 730000 Lanzhou City, Gansu Province PR China; 2 Department of Health Science Center, Northwest Minzu University, 730000 Lanzhou, Gansu China; 3 Department of oral and maxillofacial surgery, Lanzhou Stomatological Hospital, 730000 Lanzhou, Gansu China

**Keywords:** circ_0099630, TLR4, Periodontitis, miR-409-3p

## Abstract

**Background:**

Periodontitis triggers tooth loss and affects the health of population worldwide. Emerging evidence hints that circular RNAs (circRNAs) are involved in various diseases, including periodontitis. This study aimed to investigate the role of circ_0099630 in the progression of periodontitis.

**Methods:**

Periodontitis cell model was constructed by treating human periodontal ligament cells (HPDLCs) with lipopolysaccharide (LPS). Quantitative real-time PCR was used to analyze the expression of circ_0099630, microRNA-409-3p (miR-409-3p) and toll-like receptor 4 (TLR4) mRNA. Western blot was used for detecting protein levels of TLR4, cleaved-caspase 3, Bcl-2, CyclinD1 and NF-κB signaling markers. For function analyses, cell proliferation was assessed by CCK-8 assay and EdU assay. The releases of pro-inflammation factors were monitored by ELISA kits. The potential relationship between miR-409-3p and circ_0099630 or TLR4 was verified by dual-luciferase reporter assay, RIP assay and pull-down assay.

**Results:**

The expression of circ_0099630 and TLR4 was elevated in periodontitis patients and LPS-treated HPDLCs. LPS induced HPDLC proliferation inhibition, apoptosis and inflammatory responses, while circ_0099630 knockdown or TLR4 knockdown alleviated these injuries. Besides, TLR4 overexpression reversed the inhibitory effect of circ_0099630 knockdown on LPS-induced HPDLC injuries. Mechanism analysis showed that circ_0099630 positively regulated TLR4 expression by acting as miR-409-3p sponge. MiR-409-3p restoration largely ameliorated LPS-induced HPDLC injuries by depleting TLR4. Moreover, LPS activated the NF-κB signaling pathway, while circ_0099630 knockdown inhibited the activity of NF-κB signaling via the miR-409-3p/TLR4 axis.

**Conclusion:**

Circ_0099630 knockdown relieved LPS-induced HPDLC injury by miR-409-3p/TLR4 axis, suggesting that circ_0099630 might be a potential target for periodontitis treatment.

**Supplementary Information:**

The online version contains supplementary material available at 10.1186/s12903-023-03622-7.

## Introduction

Periodontitis is a chronic infectious disease of periodontal tissue, mainly affected by microorganisms in dental plaque [1]. The main characteristics of periodontitis are gum inflammation, periodontal pocket formation and alveolar bone resorption, leading to tooth loosening or loss [2, 3]. Nowadays, periodontitis is a widespread oral disease, and the incidence rate is increasing year by year worldwide [4, 5]. Increasing evidence shows that periodontitis is closely related to systemic diseases, including diabetes, hypertension, and cardiovascular diseases [6–8]. Therefore, it is important to clarify the potential pathogenesis of periodontitis to improve its treatment effects.

Periodontal ligament (PDL) tissue has the ability to stimulate periodontal regeneration, and periodontal ligament cells (PDLCs) are indispensable in the regeneration of periodontal tissue [9]. Numerous studies show that non-coding RNAs participate in the proliferation and differentiation of PDLCs in vitro, thus involving in the pathogenesis of periodontitis [10, 11]. Circular RNAs (circRNAs) are a class of non-coding RNAs, and its deregulation is closely associated with the progression of human diseases [12]. RNA-sequencing technology provided a growing number of circRNAs that were differently expressed in gingival tissues from periodontitis patients and healthy individuals [13]. According to the data, we found that circ_0099630 was highly upregulated in periodontitis patients [13]. Circ_0099630 was rarely investigated in any disorders in previous studies, and the functions of circ_0099630 were largely unknown. It was of great significance to explore the role of circ_0099630 in periodontitis to further understand the pathogenesis of periodontitis.

Toll-like receptor 4 (TLR4) is widely known as an important regulator of inflammatory responses and participates in various human inflammatory diseases [14, 15]. TLR4 was reported to be upregulated in chronic periodontitis [16, 17], and the downregulation of TLR4 contributed to the inhibition of inflammation in periodontitis [18]. Nonetheless, the mechanism of TLR4 in this disorder was not fully understood.

Accumulating studies indicate that circRNAs serve as microRNA (miRNA) sponges to be implicated in posttranscriptional regulation [19], which provides a new action mode of circRNAs. By targeting miRNAs, circRNAs can relieve the inhibition of miRNA on downstream target genes. Though bioinformatics analysis, both circ_0099630 and TLR4 3’ untranslated region (3’-UTR) harbored response elements with miR-409-3p. Therefore, we speculated that circ_0099630 might regulate TLR4 via sponging miR-409-3p and thus regulate multiple biological processes. However, the interactions between circ_0099630 and TLR4 were not addressed in previous studies.

In this study, we constructed inflammatory cell models of periodontitis by treating human periodontal ligament cells (HPDLCs) with lipopolysaccharide (LPS). We investigated the function of circ_0099630 and TLR4 on proliferation, apoptosis and inflammation of LPS-treated HPDLCs. Besides, we screened miR-409-3p and proposed the circ_0099630/miR-409-3p/TLR4 network to illustrate the functional mechanism of circ_0099630 in LPS-mediated HPDLC injuries, aiming to provide additional opinion for periodontitis treatment.

## Materials and methods

### Periodontal tissue collection

A total of 20 periodontitis patients and 20 normal controls (teeth for orthodontic) recruited at First Hospital Affiliated to Lanzhou University. After the root surface was washed with PBS, the middle 1/3 of the periodontal ligament tissues in the root was scraped and stored at -80 °C. Patients with other oral, systemic diseases and antibiotic intake within 1 month were excluded from this study. The clinicopathologic characteristics of periodontitis patients and normal controls are listed in Table 1. Written informed consent was obtained from each subject. This project was approved by the Ethics Committee of First Hospital Affiliated to Lanzhou University [Approval number: 202,111,045].

**Table 1:** The clinicopathologic characteristics of periodontitis patients and normal controls

Characteristics	Number	
	Periodontitis (N = 20)	Normal controls (N = 20)
Age (years)		
< 65	11	10
≥ 65	9	10
Sex		
Male	8	11
Female	12	9
Order of severity		
Slight + medium	13	
Server	7	
Grade of periodontitis		
Level 0 + 1	13	
Level 2	7	

Note: Level 0, individuals with a healthy periodontium and up to one proximal site with loss of attachment ≥ 3 mm; Level 1, presence of proximal attachment and loss ≥ 3 mm in ≥ 2 nonadjacent teeth; Level 2, presence of proximal attachment loss ≥ 5 mm in ≥ 30% of teeth

### Cell model construction

Healthy periodontal ligament tissues were collected from subjects underwent orthodontic treatment. HPDLCs were isolated from periodontal ligament tissues at the one third of the root of molars through the enzyme digestion. HPDLCs were cultured in DMEM (GIBCO, Grand Island, NY, USA) supplemented with 10% fetal bovine serum (FBS; GIBCO) in a 37℃ incubator containing 5% CO_2_. HPDLCs at 3 to 6 passages were used in this study.

Inflammatory cell models of periodontitis were established by treating HPDLCs with P. gingivalis LPS (1 µg/mL; Sigma-Aldrich, St. Louis, MO, USA). After time intervals of 24 h, HPDLCs were collected for the following experiments.

### Quantitative real-time PCR (qPCR)

RNA samples were isolated using TRIzol reagent (Takara, Dalian, China). For cDNA synthesis, the PrimeScript™ 1st Strand cDNA Synthesis Kit (Takara) or Mir-X miRNA First-Strand Synthesis Kit (Clontech, Mountain View, CA, USA) was used according to the protocols. Subsequently, qPCR was performed using the TB Green Fast qPCR Mix (Takara) or Mir-X miRNA TB Green Kit (Clontech) according to the protocols. We used the 2^−ΔΔCt^ method to calculate relative expression with GAPDH or U6 as an internal reference. The sequences of primers were:

circ_0099630, F: 5’-TCGGCCAAAGGAAGAATGAC-3’ and R: 5’-TTGATGAAAATGACCCATGACG-3’; miR-409-3p, F: 5’-GAATGTTGCTCGGTGAACCCCT-3’ and R: 5’-GAACATGTCTGCGTATCTC-3’; TLR4, F: 5’-AGACCTGTCCCTGAACCCTAT-3’ and R: 5’-CGATGGACTTCTAAACCAGCCA-3’; GAPDH, F: 5’-TCGGAGTCAACGGATTTGGT-3’ and R: 5’-TTCCCGTTCTCAGCCTTGAC-3’; U6, F: 5’-GCTTCGGCAGCACATATACTAAAAT-3’ and R: 5’-CGCTTCACGAATTTGCGTGTCAT-3’;

### Western blot

The procedures of western blot were accordance with the previous description [20]. Briefly, protein was extracted by RUPA buffer and quantified by BCA Kit (Beyotime, Shanghai, China). Then, 30 µg protein was separated by SDS-PAGE gel and transferred onto PVDF membranes. Membrane was blocked by non-fat milk and incubated with primary antibodies. After washing with TBST for 3 times, membrane was treated with secondary antibody. Protein signals were detected using ECL reagent (Beyotime). The primary antibodies were purchased from Abcam (Cambridge, MA, USA), including anti-TLR4 (ab13556), anti-cleaved-caspase 3 (ab2302), anti-Bcl-2 (ab692), anti-CyclinD1 (ab16663), anti-p-TAK1 (ab109404), anti-p-IκBα (ab133462), anti-p65 (ab16502) and anti-GAPDH (ab9485). The secondary antibodies were also purchased from Abcam, including goat anti-rabbit IgG (ab205718) and goat anti-mouse IgG (ab205719).

### Cell transfection

Circ_0099630-specific small interference RNA (si-circ_0099630), TLR4 overexpression fusion vector (TLR4) and their negative control (si-NC and vector) were synthesized by Genepharma (Shanghai, China). MiR-409-3p-specific mimic (miR-409-3p), inhibitor (anti-miR-409-3p) and matched negative controls (miR-NC and anti-NC) were all obtained from Ribobio (Guangzhou, China). These oligonucleotides or vectors were transfected into cells using Lipofectamine 3000 reagent (Invitrogen, Carlsbad, CA, USA).

### CCK-8 assay

After transfection, LPS-treated HPDLCs were seeded into 96-well plates and then cultured for 24 h, 48 or 72 h. At different time points, cells in each well were treated with 10 µL CCK-8 reagent (Sigma-Aldrich) for another 2 h. Cell viability was then detected using an iMARK microplate reader (Bio-Rad, Hercules, CA, USA) at 450 nm.

### EdU assay

To assess cell proliferation, EdU assay was performed using the Cell-Light EdU Apollo567 in Vitro Kit (Ribobio). After transfection, cells were treated with EdU reagent for 2 h and then fixed in PBS containing 4% paraformaldehyde for 15 min. Cells were next stained with dyeing solution for 30 min and counterstained with DAPI buffer. The positive-staining cells were observed and counted under a fluorescence microscope (Olympus, Tokyo, Japan).

### Flow cytometry assay

For cell apoptosis analysis, cells were detected by the Annexin V-FITC Apoptosis Detection Kit (Beyotime) in line with the protocol. In brief, a total of 5 × 10^4^ cells were suspended into 195 µL Annexin V-FITC binding buffer, followed by treatment with 5 µL Annexin V-FITC and 10 µL propidium iodide (PI). The apoptotic cells were detected by a flow cytometer (Beckman Coulter, Miami, FL, USA).

For cell cycle analysis, cells were detected using the Cell Cycle Analysis Kit (Beyotime) according to the protocol. In brief, cells were collected and washed with PBS. Then, cells were fixed in 70% ethanol overnight and stained with PI staining buffer (containing RNase A). The content of DNA was checked using a flow cytometer (Beckman Coulter).

### ELISA

To monitor the releases of pro-inflammatory factors, including TNF-α, IL-1β and IL-6, ELISA was carried out using TNF-α Human ELISA Kit (Invitrogen), IL-1β Human ELISA Kit (Invitrogen) and IL-6 Human ELISA Kit (Invitrogen). All procedures were accordance with the manufacturer’s instructions.

### Bioinformatics tools

The potential miRNAs interacted with circ_0099630 were predicted by circinteractome (https://circinteractome.nia.nih.gov/). The potential miRNAs interacted with TLR4-3’UR were predicted by starbase (http://starbase.sysu.edu.cn/). Circinteractome prediction methods are as follows: Open the website: https://circinteractome.nia.nih.gov/?tdsourcetag=s_pcqq_aiomsg, select “miRNA Target Sites”, input “circRNA ID: hsa_circ_0099630”, click “miRNA Target Search”. Starbase database prediction method are as below: Open the website: https://rnasysu.com/encori/index.php, select “microRNAs-Target”, click “microRNAs-mRNA”, select “Genome: the human”, select “Target Gene: TLR4”. Then, the miRNAs predicted by circinteractome and starbase database were compared, and Venn diagram analysis was performed to analyze the miRNAs that could directly combine with circ_0099630 and TLR4, respectively.

### Dual-luciferase reporter assay

The mutant sequences of circ_0099630 and TLR4-3’UTR were designed, and the wild-type (WT) and mutant-type (MUT) reporter plasmids of circ_0099630 and TLR4-3’UTR were constructed by yanzaibio Co., Ltd. (Shanghai, China), shortly naming as circ_0099630-WT, circ_0099630-MUT, TLR4-3’UTR-WT and TLR4-3’UTR-MUT. These reporter plasmids were separately transfected with miR-409-3p or miR-NC into HPDLCs. After time interval of 48 h, luciferase activity was checked using the Dual-Luciferase Reporter Assay System (Promega, Madison, WI, USA).

### RIP assay

RIP assay was performed using the Imprint RNA Immunoprecipitation Kit (Sigma-Aldrich). HPDLCs were lysed, and cell lysates were incubated with Protein A magnetic beads conjugated with human Ago2 antibody (anti-Ago2; Sigma-Aldrich) or control mouse IgG antibody (anti-IgG; Sigma-Aldrich). RNA samples bound to beads were eluted and isolated for qPCR analysis.

### Pull-down assay

Biotin-labeled miR-409-3p probe (Bio-miR-409-3p) and negative control probe (Bio-NC) were provided by Ribobio. Pull-down assay was implemented using Pierce™ Magnetic RNA-Protein Pull-Down Kit (Thermo Fisher Scientific, Waltham, MA, USA) according to the protocol. RNA compounds pulled down by Bio-miR-409-3p were eluted and isolated for qPCR analysis.

### Statistical analysis

All assays were repeated for three times to collect statistical data. The data were processed using GraphPad Prism 7 (GraphPad Software Inc., San Diego, CA, USA) and shown as the mean ± standard deviation (SD). Statistical difference in groups was compared using Student’s *t*-test (2 groups) and ANOVA followed by Tukey’s post-hoc test (multiple groups). Statistical significance was defined when *P*-value less than 0.05.

## Results

### Circ_0099630 and TLR4 were overexpressed in periodontal tissues and LPS-treated HPDLCs

**Fig. 1:**
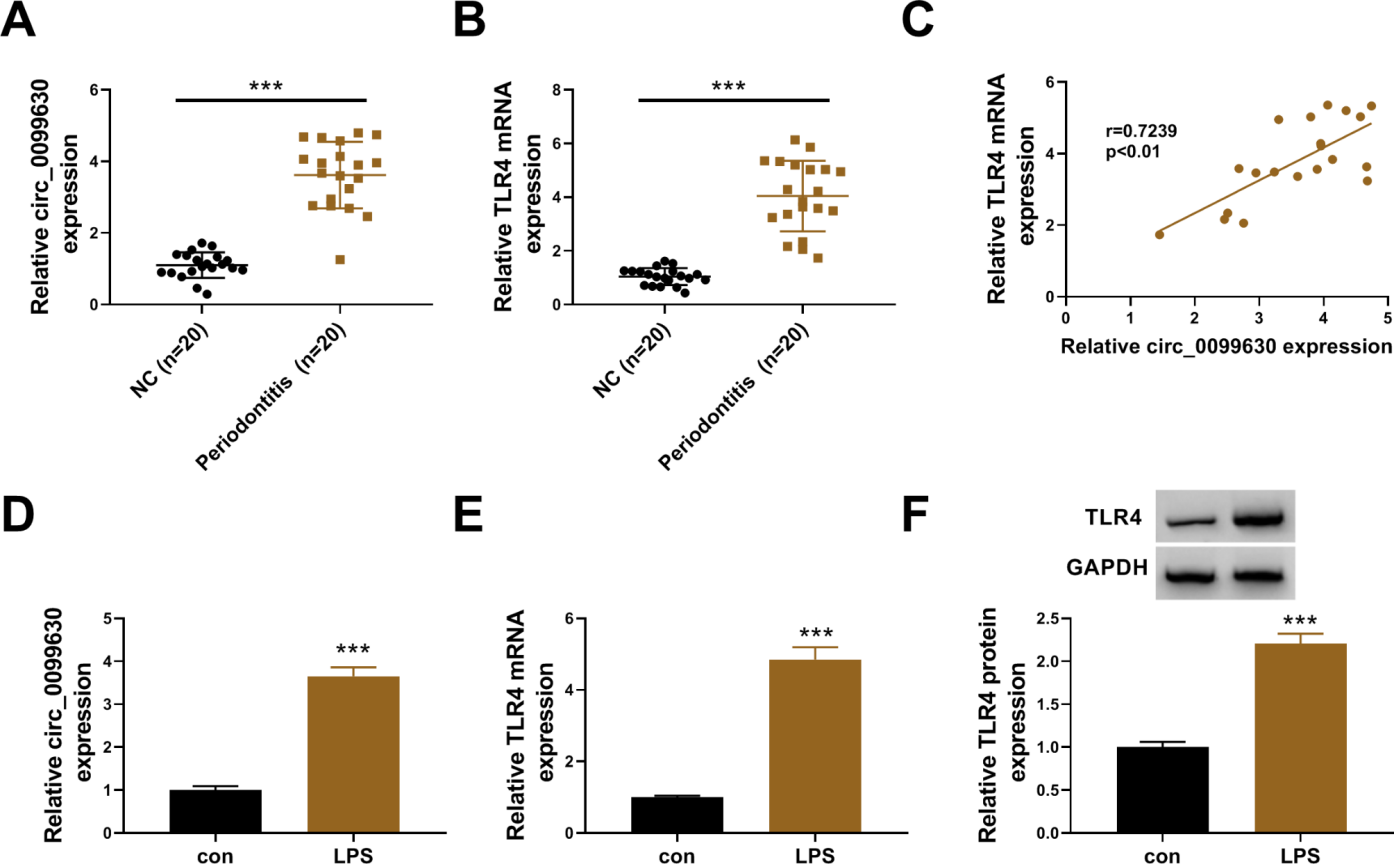
The expression of circ_0099630 and TLR4 was elevated in periodontal tissues of periodontitis and LPS-treated HPDLCs. (**A** and **B**) The expression of circ_0099630 and TLR4 in periodontal tissues from periodontitis and NC was detected by qPCR. (**C**) The correction between TLR4 expression and circ_0099630 in periodontal tissues of periodontitis was analyzed using Pearson’s analysis. (**D**) The expression of circ_0099630 in LPS-treated HPDLCs was detected by qPCR. (**E** and **F**) The expression of TLR4 mRNA and protein in LPS-treated HPDLCs was detected by qPCR and western blot. ***P < 0.001

The expression of circ_0099630 was detected in tissues and cell models at first. As shown in Fig. 1A, the expression of circ_0099630 was significantly enhanced in periodontal tissues from periodontitis patients compared to NC. Likewise, the expression of TLR4 mRNA was also significantly increased in periodontitis patients (Fig. 1B). Besides, we found that TLR4 mRNA expression was positively correlated with circ_0099630 expression in periodontal tissues of periodontitis (Fig. 1C). The expression of circ_0099630 was notably increased in LPS-treated HPDLCs (Fig. 1D), and the high TLR4 expression was observed in LPS-treated HPDLCs at both mRNA and protein levels (Fig. 1E F). The aberrant expression of circ_0099630 and TLR4 hinted that they were involved in periodontitis development.

### LPS-induced cell injuries in HPDLCs were alleviated by circ_0099630 knockdown

**Fig. 2:**
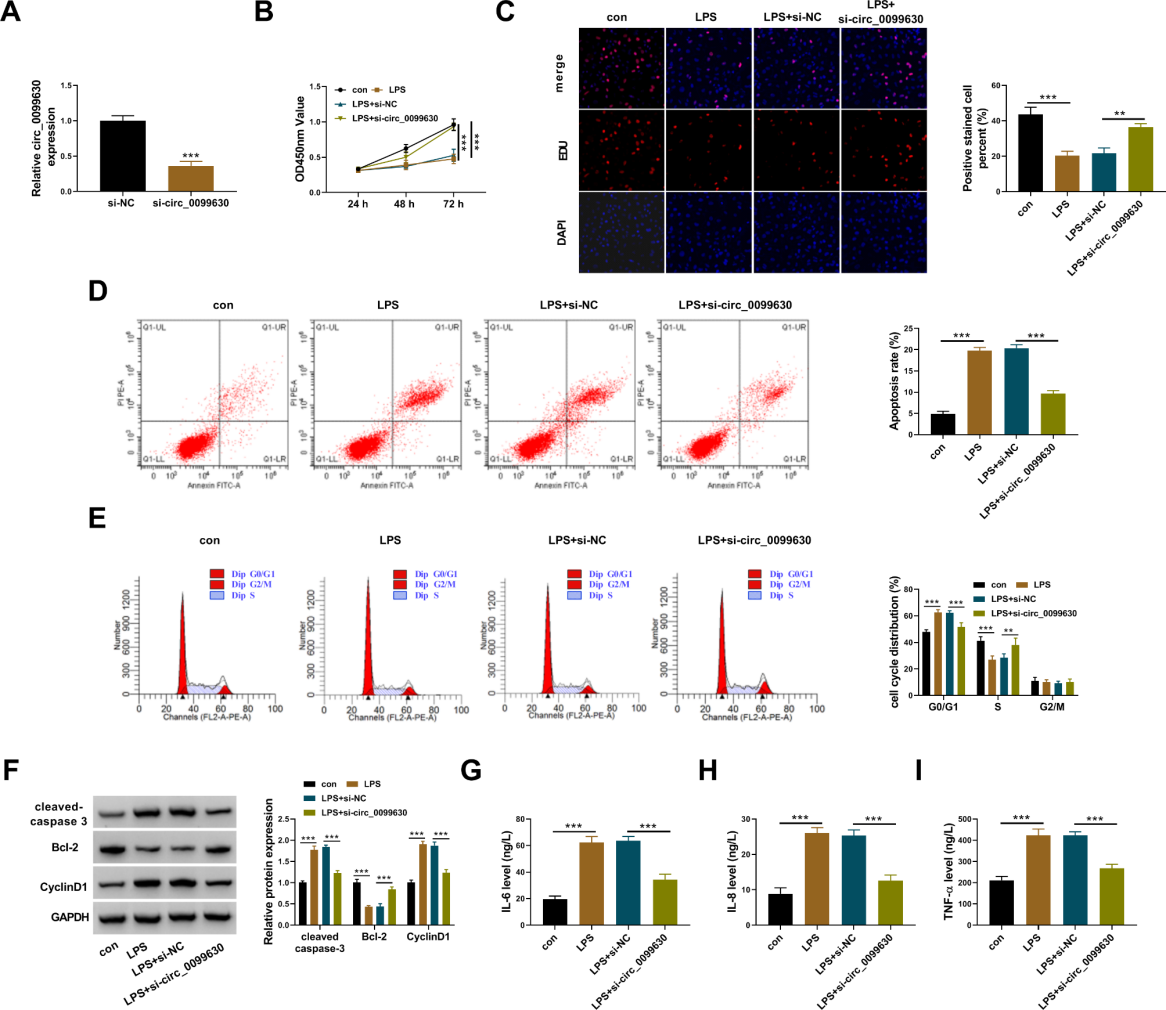
Circ_0099630 knockdown alleviated LPS-induced injuries of HPDLCs. (**A**) The expression of circ_0099630 in LPS-treated HPDLCs transfected with si-circ_0099630 or si-NC was detected by qPCR. (**B** and **C**) Cell proliferation in these experimental cells was assessed by CCK-8 assay and EdU assay. (**D** and **E**) Cell apoptosis and cell cycle progression in these experimental cells were assessed by flow cytometry assay. (**F**) The protein levels of cleaved-caspase 3, Bcl-2 and CyclinD1 in these experimental cells were detected by western blot. (**G**-**I**) The releases of IL-6, IL-8 and TNF-α in these experimental cells were checked using ELISA kits. **P < 0.01, ***P < 0.001

The expression of circ_0099630 was effectively reduced in HPDLCs by si-circ_0099630 to study the function of circ_0099630 (Fig. 2A). The capacity of cell proliferation was determined by CCK-8 assay and EdU assay. The data showed that HPDLC proliferation was largely suppressed by LPS, while circ_0099630 knockdown partly recovered this effect (Fig. 2B C). Flow cytometry assay showed that LPS-induced HPDLC apoptosis was substantially attenuated by circ_0099630 knockdown (Fig. 2D). Also, LPS significantly induced cell cycle arrest at the G0/G1 phase, while this arrest was largely alleviated by circ_0099630 knockdown (Fig. 2E). The protein levels of cleaved-caspase 3 and CyclinD1 were elevated, while the protein level of Bcl-2 was declined in LPS-induced HPDLCs. However, circ_0099630 knockdown reduced the levels of cleaved-caspase 3 and CyclinD1 but enhanced the level of Bcl-2 (Fig. 2F). Moreover, the releases of IL-6, IL-8 and TNF-α were stimulated by LPS, while circ_0099630 knockdown inhibited the releases of these pro-inflammatory factors (Fig. 2G and I). We summarized from these data that circ_0099630 knockdown alleviated LPS-induced injures of HPDLCs.

### LPS-induced cell injuries in HPDLCs were alleviated by TLR4 knockdown

**Fig. 3:**
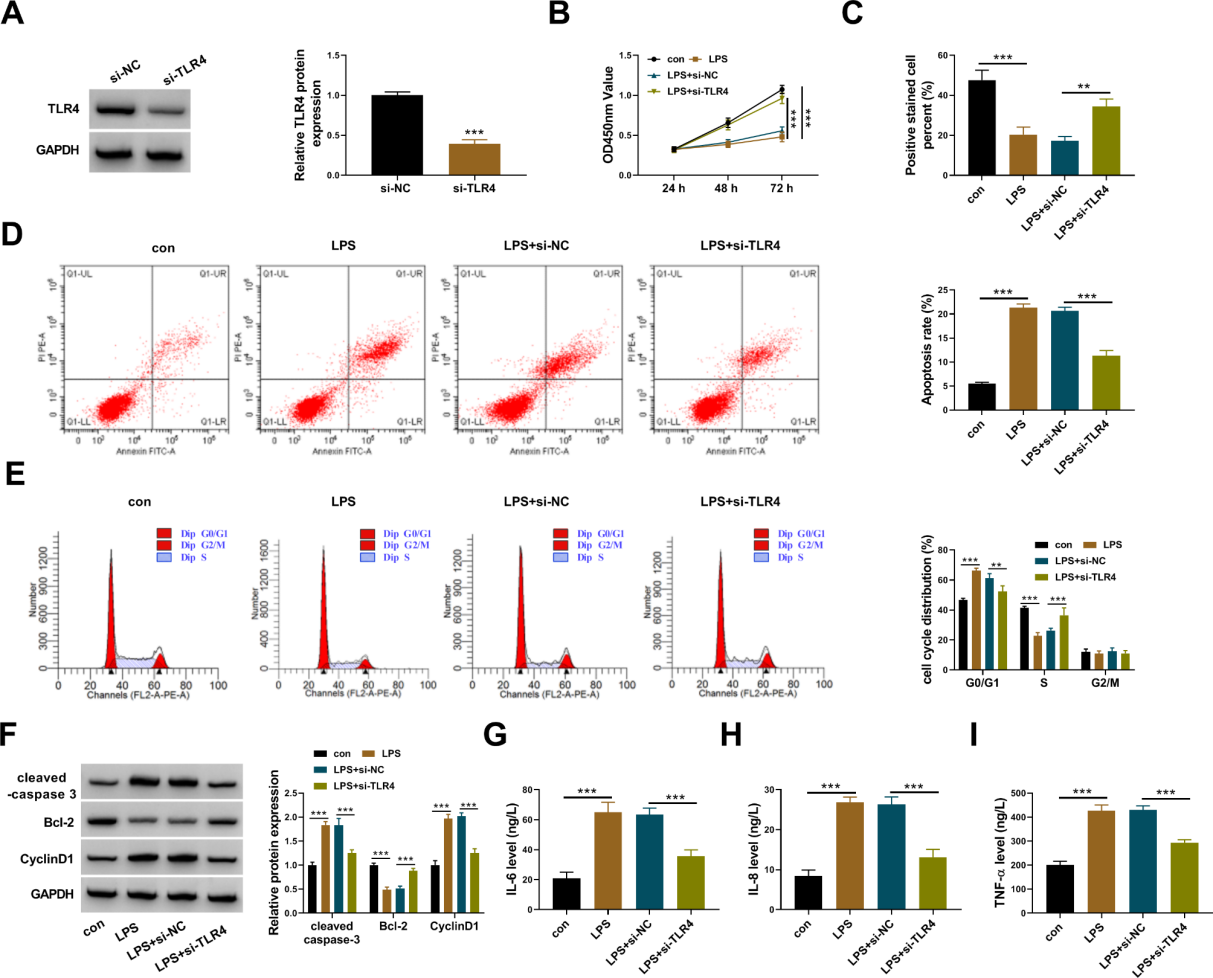
TLR4 knockdown alleviated LPS-induced injuries of HPDLCs. (**A**) The expression of TLR4 protein in LPS-treated HPDLCs transfected with si-TLR4 or si-NC was detected by western blot. (**B** and **C**) Cell proliferation in these experimental cells was assessed by CCK-8 assay and EdU assay. (**D** and **E**) Cell apoptosis and cell cycle progression in these experimental cells were assessed by flow cytometry assay. (**F**) The protein levels of cleaved-caspase 3, Bcl-2 and CyclinD1 in these experimental cells were detected by western blot. (**G**-**I**) The releases of IL-6, IL-8 and TNF-α in these experimental cells were checked using ELISA kits. **P < 0.01, ***P < 0.001

The endogenous level of TLR4 protein was also effectively decreased in HPDLCs to study the function of TLR4 (Fig. 3A). LPS-suppressed proliferative capacity of HPDLCs was largely restored by TLR4 knockdown (Fig. 3B C). In contrast, LPS-induced apoptosis rate of HPDLCs was limited by TLR4 knockdown (Fig. 3D). As for cell cycle, we discovered that TLR4 knockdown partly relieved LPS-induced cell cycle arrest (Fig. 3E). The protein levels of cleaved-caspase 3 and CyclinD1 enhanced by LPS administration were substantially reduced by TLR4 downregulation, while the protein level of Bcl-2 inhibited by LPS administration was substantially enhanced by TLR4 knockdown (Fig. 3F). The releases of IL-6, IL-8 and TNF-α were stimulated by LPS but largely suppressed by TLR4 knockdown (Fig. 3G and I). The data hinted that TLR4 knockdown alleviated LPS-induced cell injuries of HPDLCs.

### TLR4 overexpression reversed the effects of circ_0099630 knockdown in LPS-treated HPDLCs

**Fig. 4:**
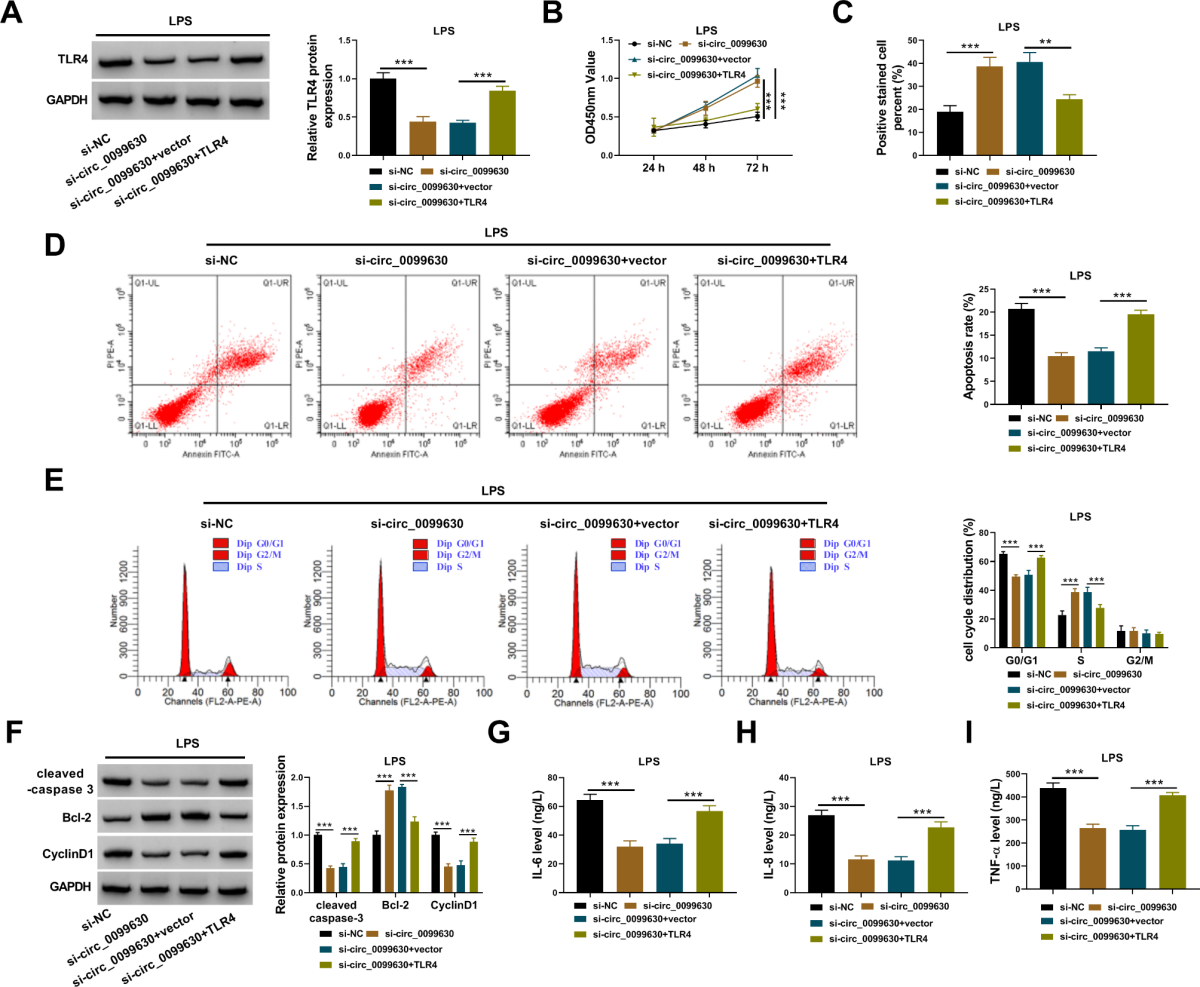
TLR4 overexpression reversed the role of circ_0099630 knockdown and recovered LPS-induced injuries of HPDLCs. (**A**) The expression of TLR4 protein in LPS-treated HPDLCs transfected with si-circ_0099630 alone or si-circ_0099630 + TLR4 was detected by western blot. In these transfected cells, (**B** and **C**) cell proliferation was assessed by CCK-8 assay and EdU assay. (**D** and **E**) Cell apoptosis and cell cycle distribution were monitored by flow cytometry assay. (**F**) The protein levels of cleaved-caspase 3, Bcl-2 and CyclinD1 were detected by western blot. (**G**-**I**) The releases of IL-6, IL-8 and TNF-αwere checked using ELISA kits. **P < 0.01, ***P < 0.001

The protein level of TLR4 was strikingly decreased in LPS-treated HPDLCs transfected with si-circ_0099630, while the protein level of TLR4 was largely recovered in LPS-treated HPDLCs transfected with si-circ_0099630 + TLR4 (Fig. 4A). In function, cell proliferative capacity was largely recovered in LPS-treated HPDLCs transfected with si-circ_0099630 but repressed in LPS-treated HPDLCs transfected with si-circ_0099630 + TLR4 (Fig. 4B C). The apoptosis rate of HPDLCs was alleviated by alone circ_0099630 knockdown, while additional TLR4 overexpression promoted the apoptosis rate of HPDLCs (Fig. 4D). TLR4 overexpression substantially induced cell cycle arrest that was alleviated by circ_0099630 knockdown alone (Fig. 4E). The expression of cleaved-caspase 3 and CyclinD1 was reduced in LPS-treated HPDLCs transfected with si-circ_0099630 but recovered in cells transfected with si-circ_0099630 + TLR4, while the expression of Bcl-2 was enhanced by si-circ_0099630 but repressed by circ_0099630 + TLR4 in LPS-treated HPDLCs (Fig. 4F). The releases of IL-6, IL-8 and TNF-α in LPS-treated HPDLCs were significantly blocked by si-circ_0099630 transfection but promoted by si-circ_0099630 + TLR4 transfection (Fig. 4G and I). These findings indicated that circ_0099630 knockdown alleviated LPS-induced injures of HPDLCs by downregulating TLR4.

### Circ_0099630 functioned as Mir-409-3p sponge to regulate TLR4 expression

**Fig. 5:**
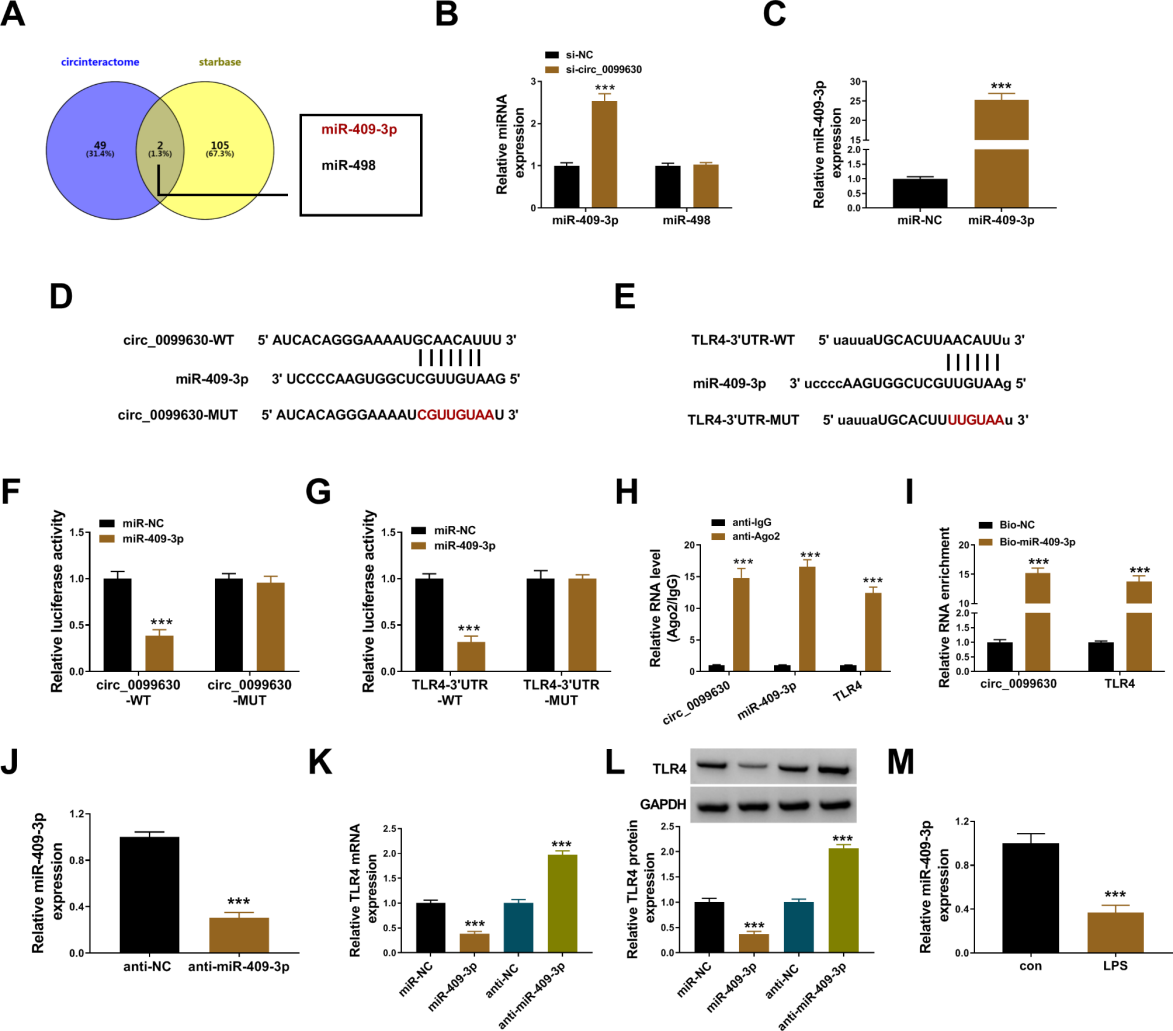
Circ_0099630 and TLR4 3’UTR harbored response elements with miR-409-3p. (**A**) The target miRNAs of circ_0099630 were predicted by circinteractome, and the potential miRNAs interacted with TLR4 were predicted by starbase. (**B**) The expression of miR-409-3p and miR-498 in HPDLCs with circ_0099630 knockdown was detected by qPCR. (**C**) The expression of miR-498 in HPDLCs transfected with miR-498 mimic was detected by qPCR. (**D** and **E**) The WT and MUT sequence fragments of circ_0099630 and TLR4 3’UTR. (**F** and **G**) The relationship between miR-409-3p and circ_0099630 or TLR4 was verified by dual-luciferase reporter assay. (**H**) The relationship between miR-409-3p and circ_0099630 or TLR4 was verified by RIP assay. (**I**) The relationship between miR-409-3p and circ_0099630 or TLR4 was verified by pull-down assay. (**J**) The expression of miR-409-3p in HPDLCs transfected with anti-miR-409-3p or anti-NC was detected by qPCR. (**K** and **L**) The expression of TLR4 mRNA and protein in HPDLCs with miR-409-3p restoration or deficiency was detected by qPCR and western blot. (**M**) The expression of miR-409-3p in LPS-treated HPDLCs was detected by qPCR. ***P < 0.001

To explore the interaction between circ_0099630 and TLR4, we screened miRNAs that harbored response elements with both circ_0099630 and TLR4. The analysis from circinteractome and starbase showed that only 2 miRNAs (miR-409-3p and miR-498) were found to harbor binding sites with both circ_0099630 and TLR4 (Fig. 5A). In HPDLCs with circ_0099630 knockdown, the expression of miR-409-3p was notably increased, while miR-198 expression was rarely changed (Fig. 5B). We chose miR-409-3p for the following assays. The expression of miR-409-3p was strikingly enhanced in cells transfected with miR-409-3p mimic (Fig. 5C). For dual-luciferase reporter assay, the WT and MUT reporter plasmids of circ_0099630 and TLR4-3’UTR were constructed, and the sequences were shown in Fig. 5D and E. Dual-luciferase reporter assay presented that luciferase activity was significantly declined in HPDLCs transfected with miR-409-3p and circ_0099630-WT plasmid or TLR4-3’UTR-WT plasmid (Fig. 5F and G). RIP assay presented that circ_0099630, miR-409-3p and TLR4 were abundantly detected in the anti-Ago2-enriched compounds compared to anti-IgG (Fig. 5H). Pull-down assay presented that circ_0099630 and TLR4 could be abundantly enriched by Bio-miR-409-3p probe compared to Bio-NC (Fig. 5I). The expression of miR-409-3p was strikingly reduced in cells transfected with anti-miR-409-3p (Fig. 5J). The expression of TLR4 mRNA and protein was notably suppressed in HPDLCs transfected with miR-409-3p compared to miR-NC, while the expression of TLR4 was notably elevated in HPDLCs transfected with anti-miR-409-3p compared to anti-NC (Fig. 5K L). In addition, the expression of miR-409-3p was shown to be decreased in LPS-treated HPDLCs (Fig. 5M). These data suggested that circ_0099630 functioned as miR-409-3p sponge to regulate TLR4 expression.

#### MiR-409-3p restoration alleviated LPS-induced cell injuries of HPDLCs

**Fig. 6:**
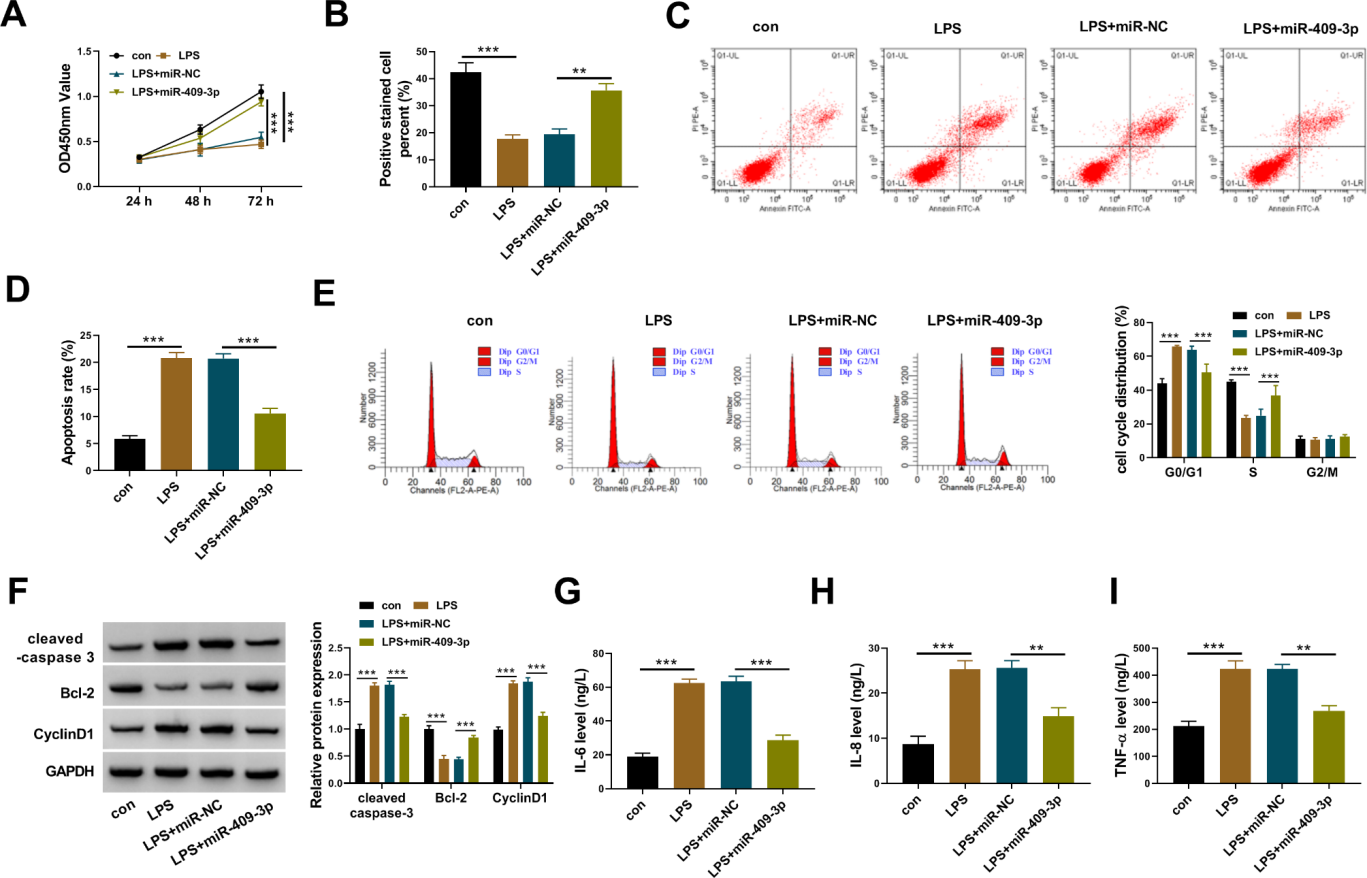
MiR-409-3p restoration alleviated LPS-induced injuries of HPDLCs. In LPS-treated HPDLCs transfected with miR-409-3p or miR-NC, (**A** and **B**) Cell proliferation in these experimental cells was assessed by CCK-8 assay and EdU assay. (**C**-**E**) Cell apoptosis and cell cycle progression in these experimental cells were assessed by flow cytometry assay. (**F**) The protein levels of cleaved-caspase 3, Bcl-2 and CyclinD1 in these experimental cells were detected by western blot. (**G**-**I**) The releases of IL-6, IL-8 and TNF-α in these experimental cells were checked using ELISA kits. **P < 0.01, ***P < 0.001

We next investigated the function of miR-409-3p in LPS-treated HPDLCs. LPS-impaired cell proliferative capacity was largely recovered by miR-409-3p restoration (Fig. 6A and B). LPS-induced cell apoptosis of HPDLCs was partially suppressed by miR-409-3p restoration (Fig. 6C and D). Besides, LPS-induced cell cycle arrest was also notably alleviated by miR-409-3p restoration (Fig. 6E). The protein levels of cleaved-caspase 3 and CyclinD1 were inhibited by miR-409-3p, while the protein level of Bcl-2 was enhanced by miR-409-3p in LPS-treated HPDLCs (Fig. 6F). Moreover, the releases of IL-6, IL-8 and TNF-α stimulated by LPS were largely suppressed in LPS-treated HPDLCs transfected with miR-409-3p (Fig. 6G and I). The data suggested that LPS-induced cell injuries of HPDLCs were also alleviated by miR-409-3p restoration.

### TLR4 overexpression reversed the effects of mir-409-3p restoration in LPS-treated HPDLCs

**Fig. 7:**
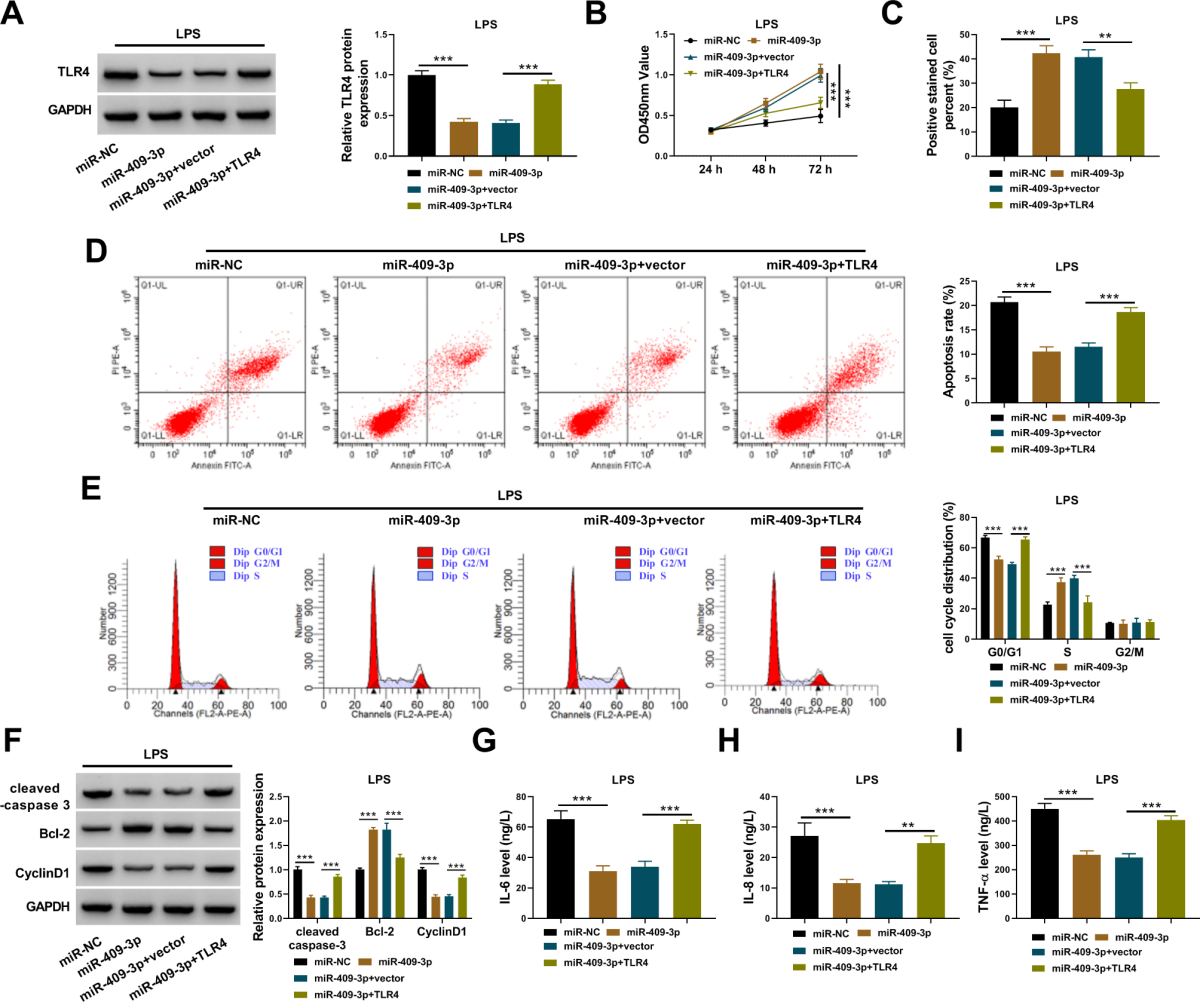
TLR4 overexpression reversed the role of miR-409-3p restoration and recovered LPS-induced injuries of HPDLCs. (**A**) The expression of TLR4 protein in LPS-treated HPDLCs transfected with miR-409-3p alone or miR-409-3p + TLR4 was detected by western blot. In these transfected cells, (**B** and **C**) cell proliferation was assessed by CCK-8 assay and EdU assay. (**D** and **E**) Cell apoptosis and cell cycle distribution were monitored by flow cytometry assay. (**F**) The protein levels of cleaved-caspase 3, Bcl-2 and CyclinD1 were detected by western blot. (**G**-**I**) The releases of IL-6, IL-8 and TNF-αwere checked using ELISA kits. **P < 0.01, ***P < 0.001

The expression of TLR4 protein was significantly declined in LPS-treated HPDLCs transfected with miR-409-3p, while the expression of TLR4 was largely recovered in LPS-treated HPDLCs transfected with miR-409-3p + TLR4 (Fig. 7A). In terms of function, cell proliferative capacity was notably recovered by miR-409-3p restoration but largely repressed by TLR4 overexpression in LPS-treated HPDLCs (Fig. 7B C). Cell apoptosis was largely blocked in LPS-treated HPDLCs transfected with miR-409-3p but partially enhanced in cells transfected with miR-409-3p + TLR4 (Fig. 7D). Cell cycle arrest in LPS-treated HPDLCs was largely alleviated by miR-409-3p transfection, while cell cycle was substantially arrested at the G0/G1 phase by miR-409-3p + TLR4 transfection (Fig. 7E). The expression of cleaved-caspase 3 and CyclinD1 was notably suppressed in LPS-treated HPDLCs transfected with miR-409-3p but recovered in cells transfected with miR-409-3p + TLR4, while the expression of Bcl-2 was opposite to them (Fig. 7F). The releases of IL-6, IL-8 and TNF-α were markedly blocked in LPS-treated HPDLCs transfected with miR-409-3p but accelerated in LPS-treated HPDLCs transfected with miR-409-3p + TLR4 (Fig. 7G and I). These data indicated that miR-409-3p restoration alleviated LPS-induced HPDLC injuries by degrading TLR4.

#### NF-κB signaling pathway activated in LPS-treated HPDLCs was modulated by the circ_0099630/miR-409-3p/TLR4 axis

**Fig. 8:**
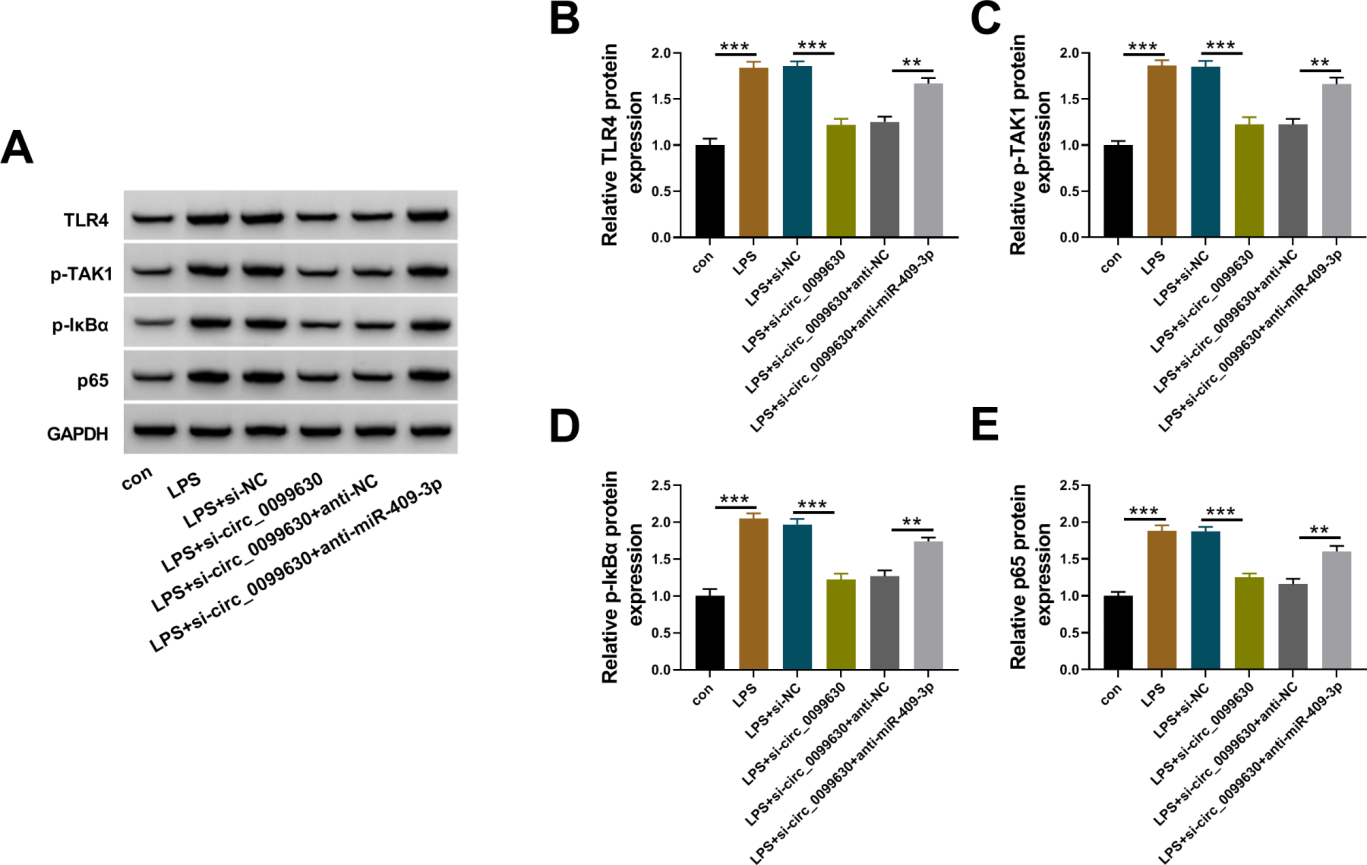
Circ_0099630 knockdown inhibited the activity of NF-κB signaling pathway via the miR-409-3p/TLR4 axis in LPS-treated HPDLCs. (**A**) Western blot signals of TLR4, p-TAK1, p-IκBα and p65 proteins in LPS-treated HPDLCs, LPS-treated HPDLCs transfected with si-circ_0099630 or si-circ_0099630 + anti-miR-409-3p were shown. (**B**-**E**) The relative expression of TLR4, p-TAK1, p-IκBα and p65 proteins in these cells was quantified from western blot. **P < 0.01, ***P < 0.001

NF-κB signaling pathway was documented to be activated in various human diseases. Interestingly, we found that TLR4 expression was inhibited by si-circ_0099630 in LPS-treated HPDLCs but recovered by si-circ_0099630 + anti-miR-409-3p (Fig. 8A and B). The protein levels of p-TAK1, p-IκBα and p65 were shown to be increased in LPS-induced HPDLCs, while si-circ_0099630 transfection weakened the protein levels of p-TAK1, p-IκBα and p65. In addition, the protein levels of p-TAK1, p-IκBα and p65 were partially recovered in LPS-induced HPDLCs with si-circ_0099630 + anti-miR-409-3p transfection (Fig. 8A C-8E). The data hinted that circ_0099630 knockdown alleviated LPS-induced HPDLC injuries by inhibiting the activity of the NF-κB signaling pathway by targeting the miR-409-3p/TLR4 axis.

## Discussion

The present study explored the role and mechanism of circ_0099630 in periodontitis. We mainly discovered that the expression of circ_0099630 and TLR4 was elevated in periodontitis patients and LPS-treated HPDLCs. Knockdown of circ_0099630 or TLR4 partially restored LPS-induced HPDLCs proliferation inhibition, apoptosis and inflammation. In addition, we for the first time proposed the circ_0099630/miR-409-3p/TLR4 network to illustrate the new mechanism of circ_0099630 in periodontitis.

HPDLCs are responsible for tooth support, collagen production and tissue regeneration [21]. LPS is the major component of the cell wall of gram-negative bacteria. Periodontitis bacteria, especially LPS, is widely shown to induce HPDLCs to secrete pro-inflammatory factors, thus playing a key role in the progression of periodontitis [22, 23]. In our study, we constructed cell models of periodontitis by treating HPDLCs with LPS. At present, circ_0099630 was only reported to be upregulated in periodontitis tissues through the data of circRNA expression profiles [13]. To verify this result, we checked the expression of circ_0099630 in clinical periodontal tissues and found that circ_0099630 was also highly expressed in periodontitis patients. Besides, circ_0099630 knockdown alleviated LPS-induced proliferation inhibition, apoptosis and inflammatory responses of HPDLCs, suggesting that circ_0099630 might promote periodontitis progression.

Toll-like receptors (TLRs) are involved in the immune response by recognizing patterns presented by bacteria and other pathogens [24]. TLR4 has been shown to be closely implicated in inflammatory responses that caused gingivitis and periodontitis [25]. TLR4 was upregulated in LPS-treated HPDLCs, and tripartite motif-containing 52 (TRIM52) knockdown alleviated LPS-induced inflammatory responses by inhibiting TLR4 [26]. The expression of TLR4 was also elevated in the experimental periodontitis rat, and Baicalin played anti-inflammatory effects in periodontitis rat by depleting the expression of TLR4 [27]. Consistent with these studies, we showed that TLR4 was upregulated in periodontitis patients and LPS-treated HPDLCs. TLR4 knockdown attenuated LPS-induced HPDLC proliferative inhibition, apoptosis and inflammatory responses. Interestingly, we found that TLR4 overexpression could reversed the effects of circ_0099630 knockdown and recovered LPS-induced injuries in HPDLCs, confirming that TLR4 might be a potential target for periodontitis treatment.

To realize the interactions between circ_0099630 and TLR4, we screened potential miRNAs that harbored binding sites with both circ_0099630 and TLR4 3’-UTR. As a result, the target relationship between miR-409-3p and circ_0099630 or TLR4 was confirmed by dual-luciferase reporter assay, RIP assay and pull-down assay. MiR-409-3p has been reported to play crucial roles in various human diseases and cancers [28, 29]. However, the role of miR-409-3p in periodontitis is still unknown. Our study discovered that miR-409-3p restoration relieved LPS-induced proliferation inhibition, apoptosis and inflammation in HPDLCs, while TLR4 overexpression abolished these effects, suggesting that miR-409-3p alleviated LPS-induced injuries in HPDLCs by sequestering TLR4. Moreover, TLR4 expression was notably decreased in HPDLCs with circ_0099630 knockdown, while additional miR-409-3p deficiency recovered TLR4 expression. We summarized that circ_0099630 positively regulated TLR4 expression by serving as miR-409-3p sponge.

By reviewing the previous studies, we found that the NF-κB signaling pathway was one of the downstream pathways of TLR4 [30]. It was reported that TLR4 activated the NF-κB signaling pathway to induce the release of pro-inflammatory factors [31], and Resveratrol played protective effects against experimental periodontitis in mice via inhibiting the phosphorylation of NF-κB signaling molecules, including p65, p38MAPK, and STAT3 [32]. Similarly, TRIM52 knockdown inhibited the release of pro-inflammatory factors by suppressing TLR4 expression and the phosphorylation of downstream NF-κB signaling molecules, including TAK1, IKK-α/β, IκBα and p65 [26]. In our study, we found that the phosphorylation of TAK1, IκBα and p65 was activated in LPS-treated HPDLCs, while circ_0099630 knockdown weakened the phosphorylation of these molecules. The data hinted that circ_0099630 knockdown inhibited the activity of the NF-κB signaling pathway through the miR-409-3p/TLR4 axis, thus alleviating LPS-induced injuries in HPDLCs.

Collectively, circ_0099630 and TLR4 were highly expressed in periodontitis patients and LPS-treated HPDLCs. Circ_0099630 knockdown alleviated LPS-induced dysfunctions and injuries of HPDLCs through miR-409-3p-mediated TLR4 inhibition. Besides, circ_0099630 knockdown might regulate the inhibition of the NF-κB signaling pathway via the miR-409-3p/TLR4 axis, which needed further verification. Our study provided a new insights into the understanding of periodontitis development.

### Electronic supplementary material

Below is the link to the electronic supplementary material.

Supplementary Material 1

Supplementary Material 2

## Data Availability

Data are however available from the authors upon reasonable request.
