# Supplementary material for: Circ_0099630 knockdown alleviates lipopolysaccharide-induced injuries of human periodontal ligament cells through the inhibition of TLR4 by releasing miR-409-3p
Source: BMC Oral Health. 2023 Nov 25;23:922. doi: 10.1186/s12903-023-03622-7 (PMC10675886; doi:10.1186/s12903-023-03622-7)

Fig 1F

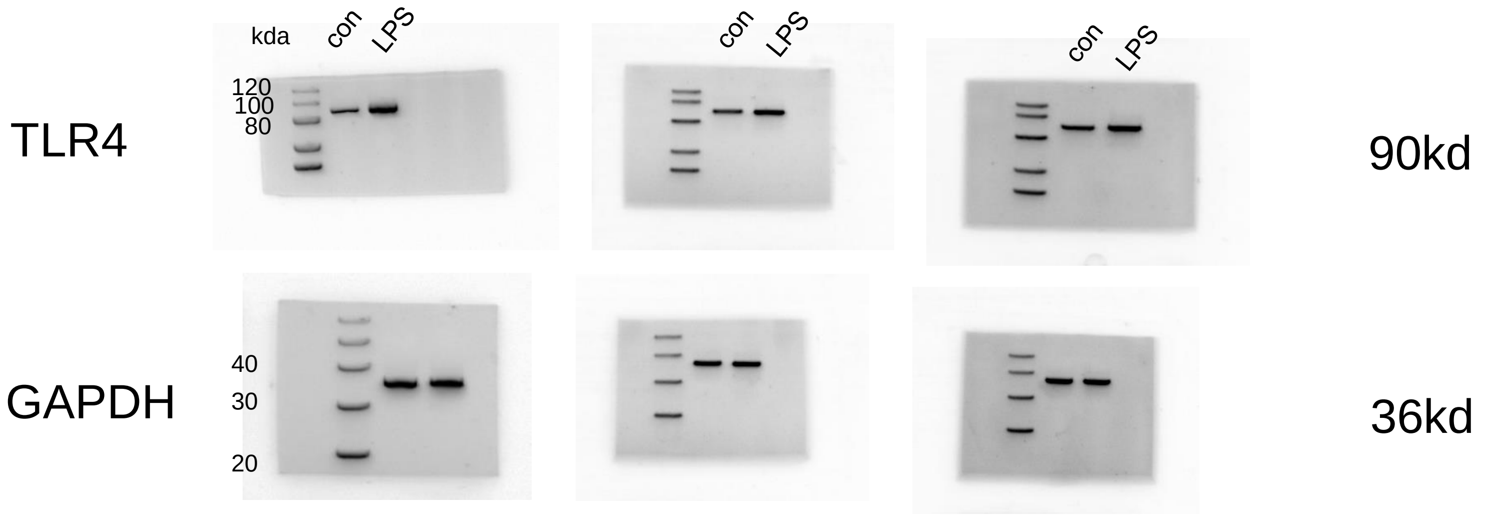
kda
con
LPS
120
100
80
TLR4
con
LPS
con
LPS
90kd
GAPDH
40
30
20
36kd

Fig 2F

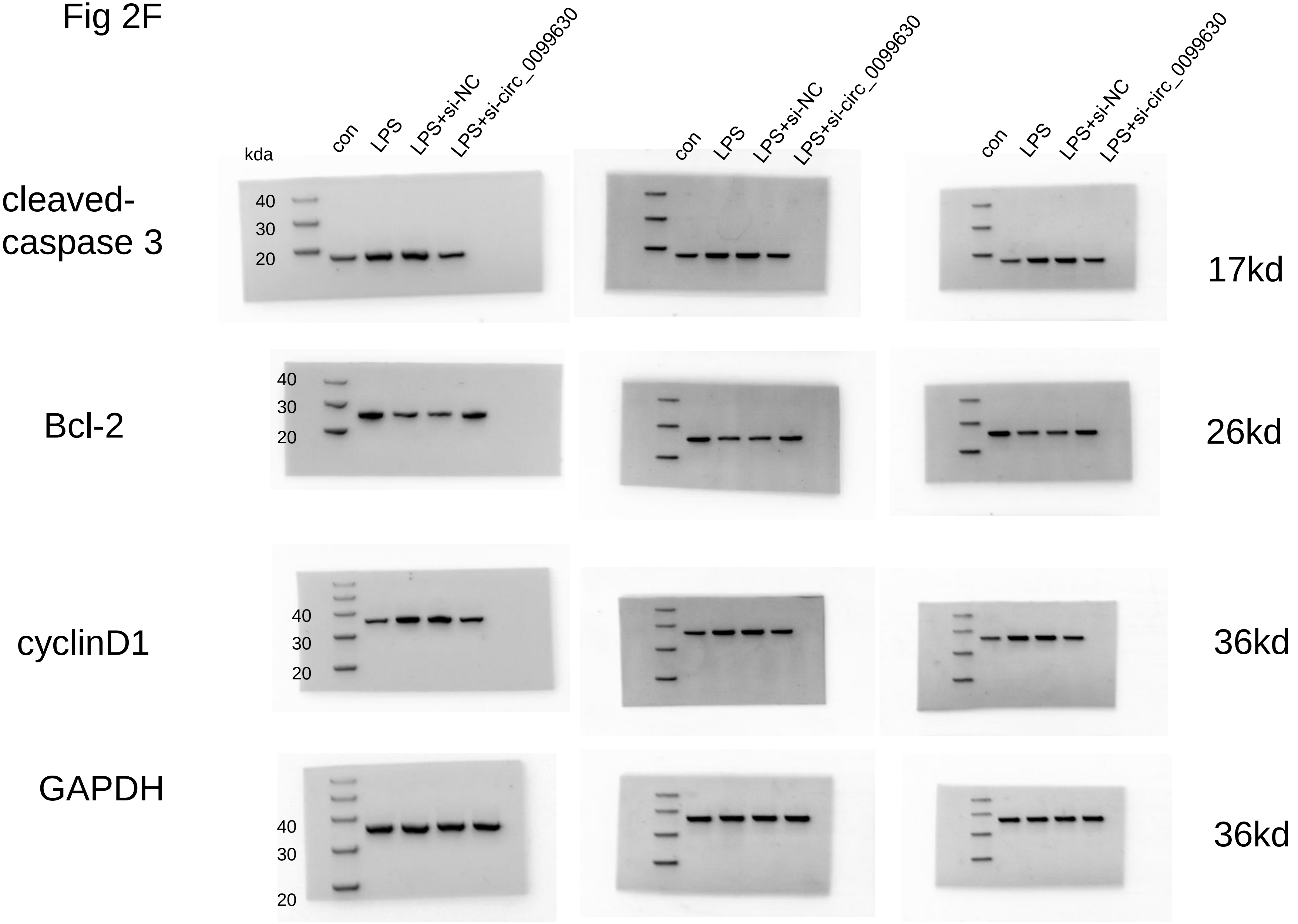
con
LPS
LPS+si-NC
LPS+si-circ_0099630
kda
40
30
20
cleaved-caspase 3
17kd
Bcl-2
26kd
cyclinD1
36kd
GAPDH
36kd

Fig 3A

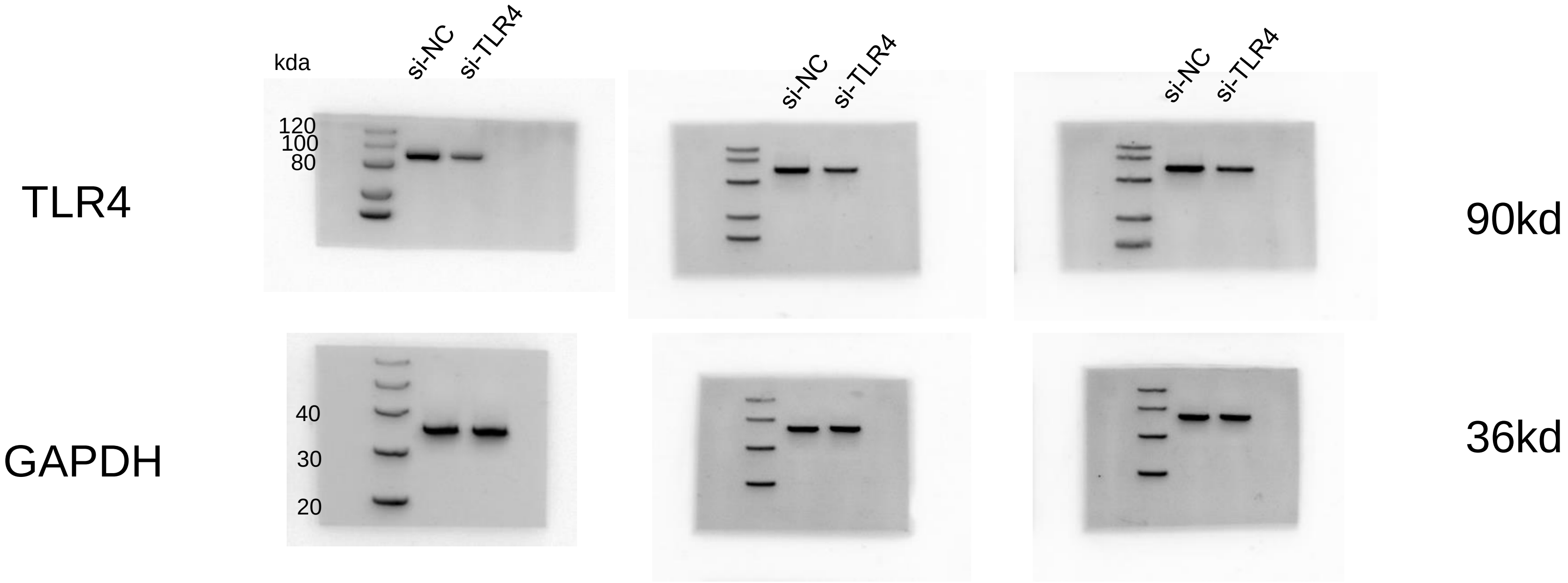

Fig 3F

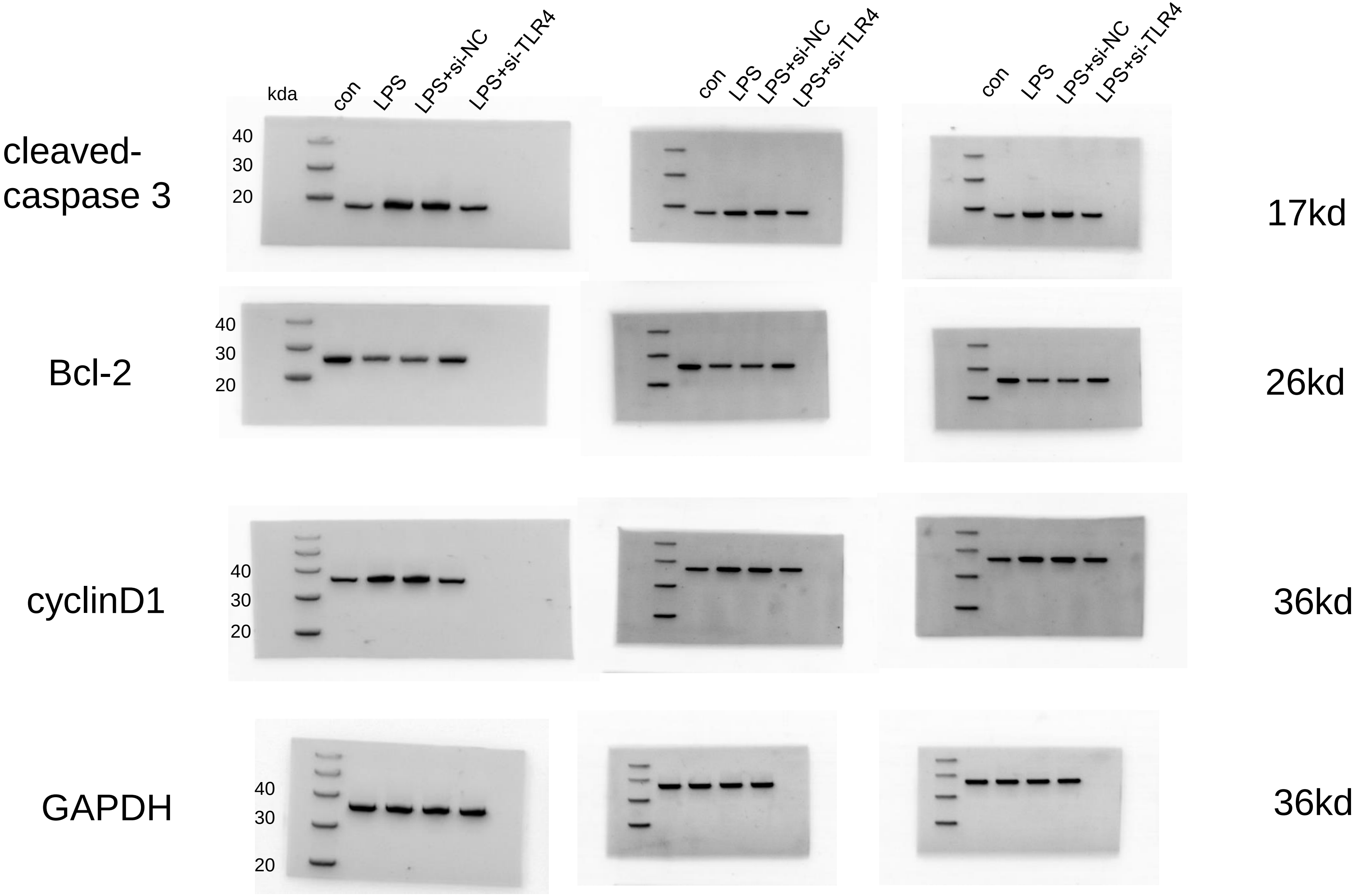
kda
con
LPS
LPS+si-NC
LPS+si-TLR4
40
30
20
cleaved-caspase 3
17kd
Bcl-2
26kd
cyclinD1
36kd
GAPDH
36kd

Fig 4A

1: LPS+si-NC 2: LPS+si-circ_0099630 3: LPS+si-circ_0099630+vector

4: LPS+si-circ_0099630+TLR4

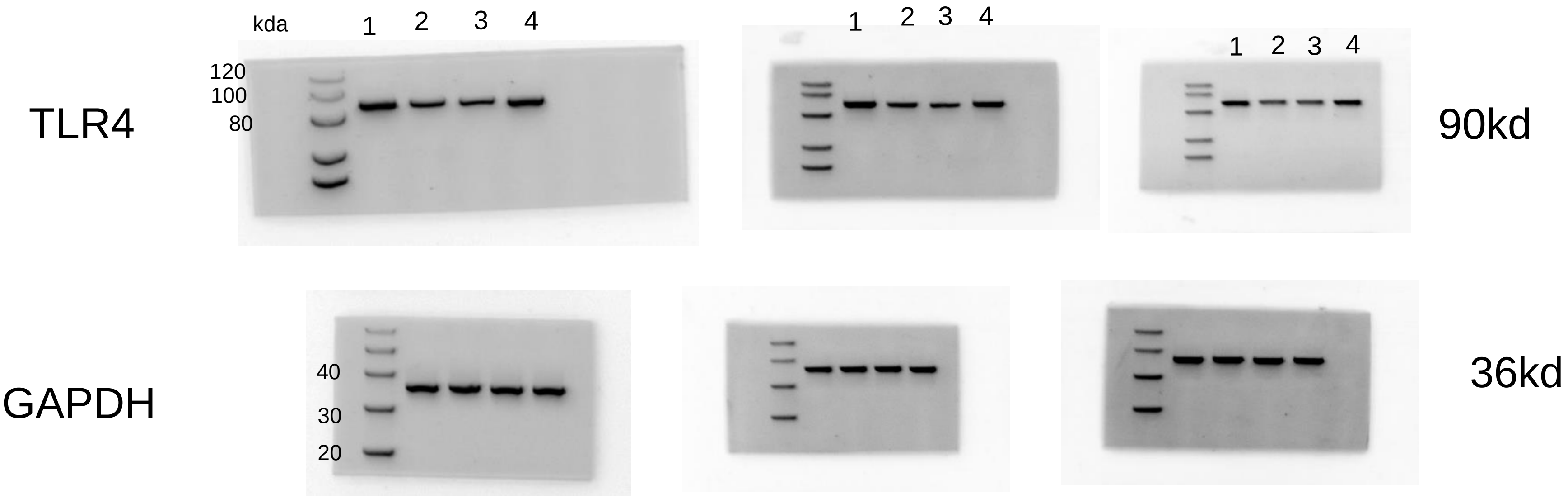

Fig 4F

1: LPS+si-NC 2: LPS+si-circ_0099630 3: LPS+si-circ_0099630+vector

4: LPS+si-circ_0099630+TLR4

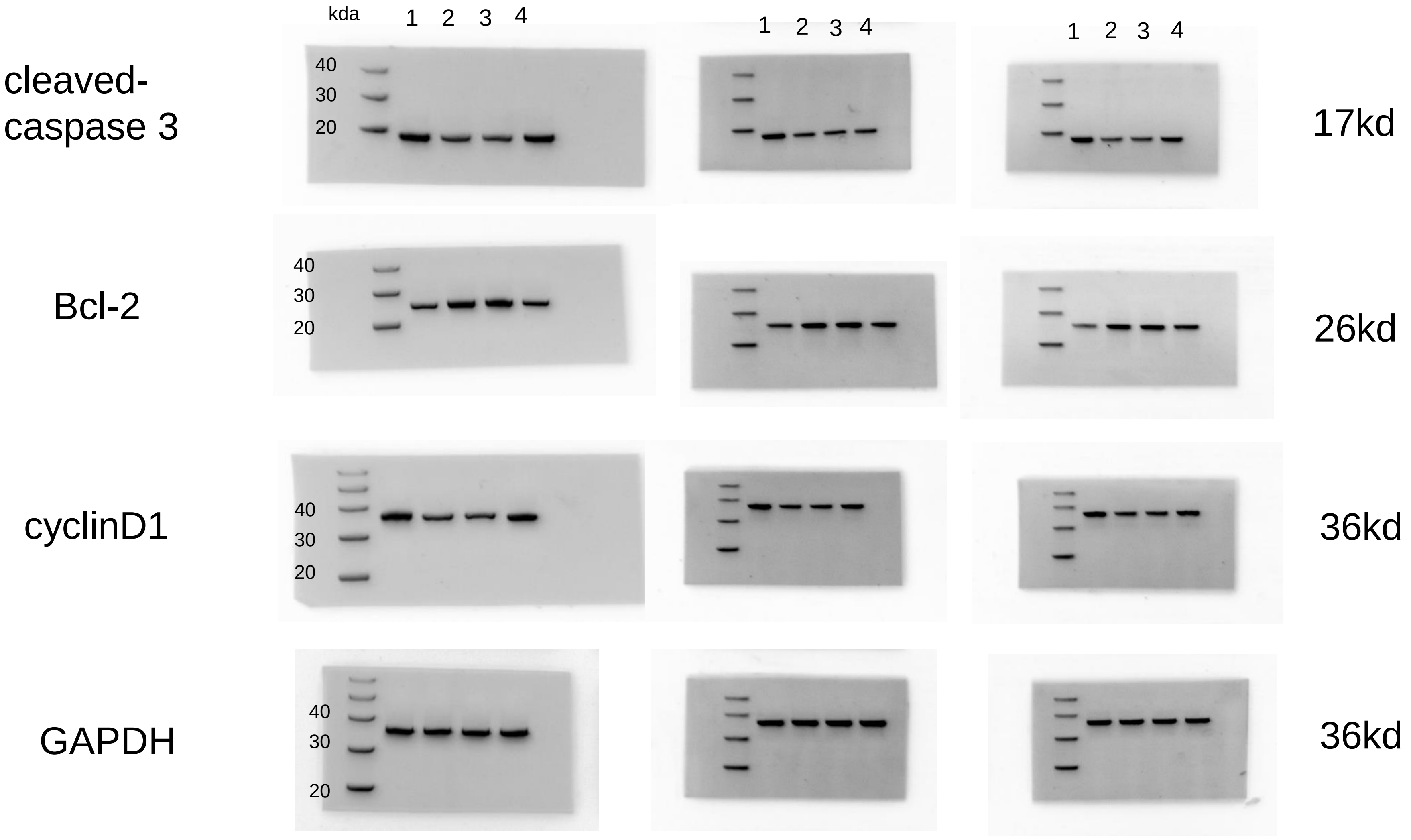

Fig 5L

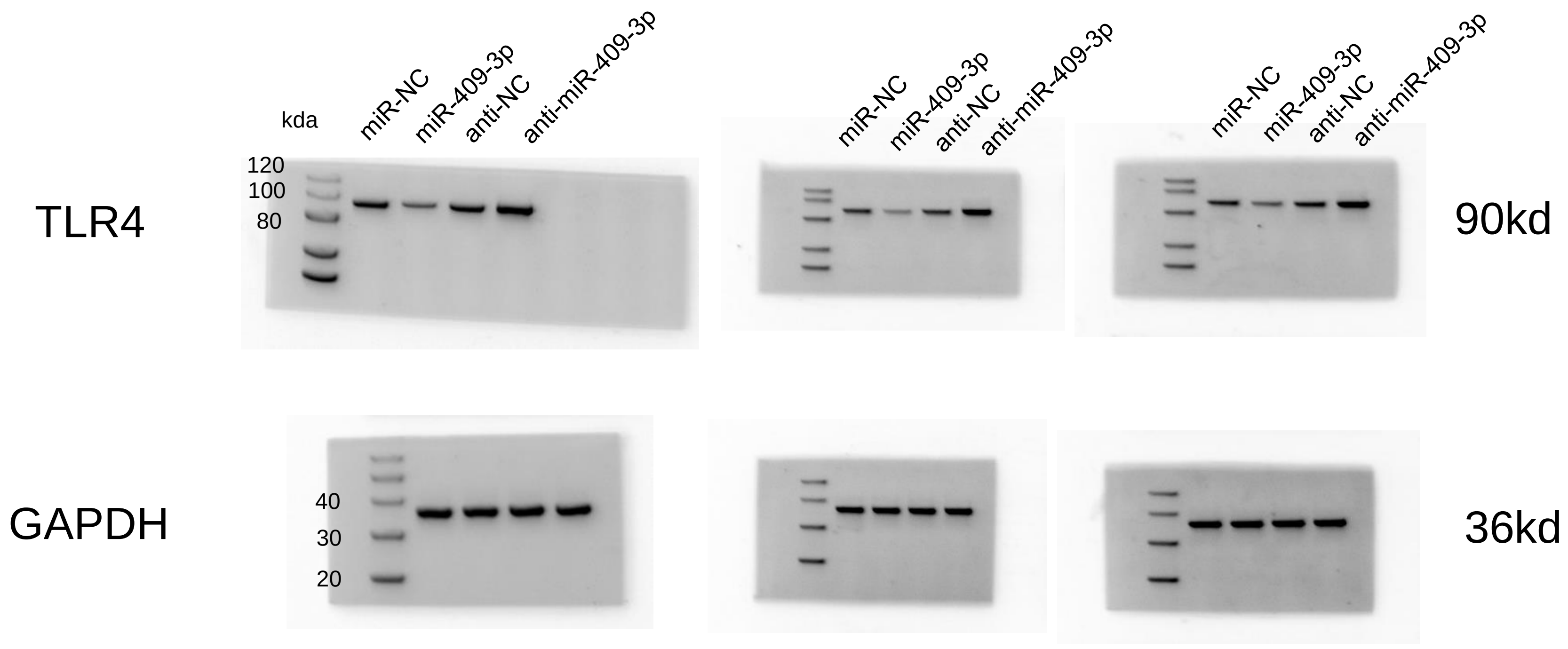
kda
miR-NC
miR-409-3p
anti-NC
anti-miR-409-3p
120
100
80
TLR4
90kd
40
30
20
GAPDH
36kd

Fig 6F

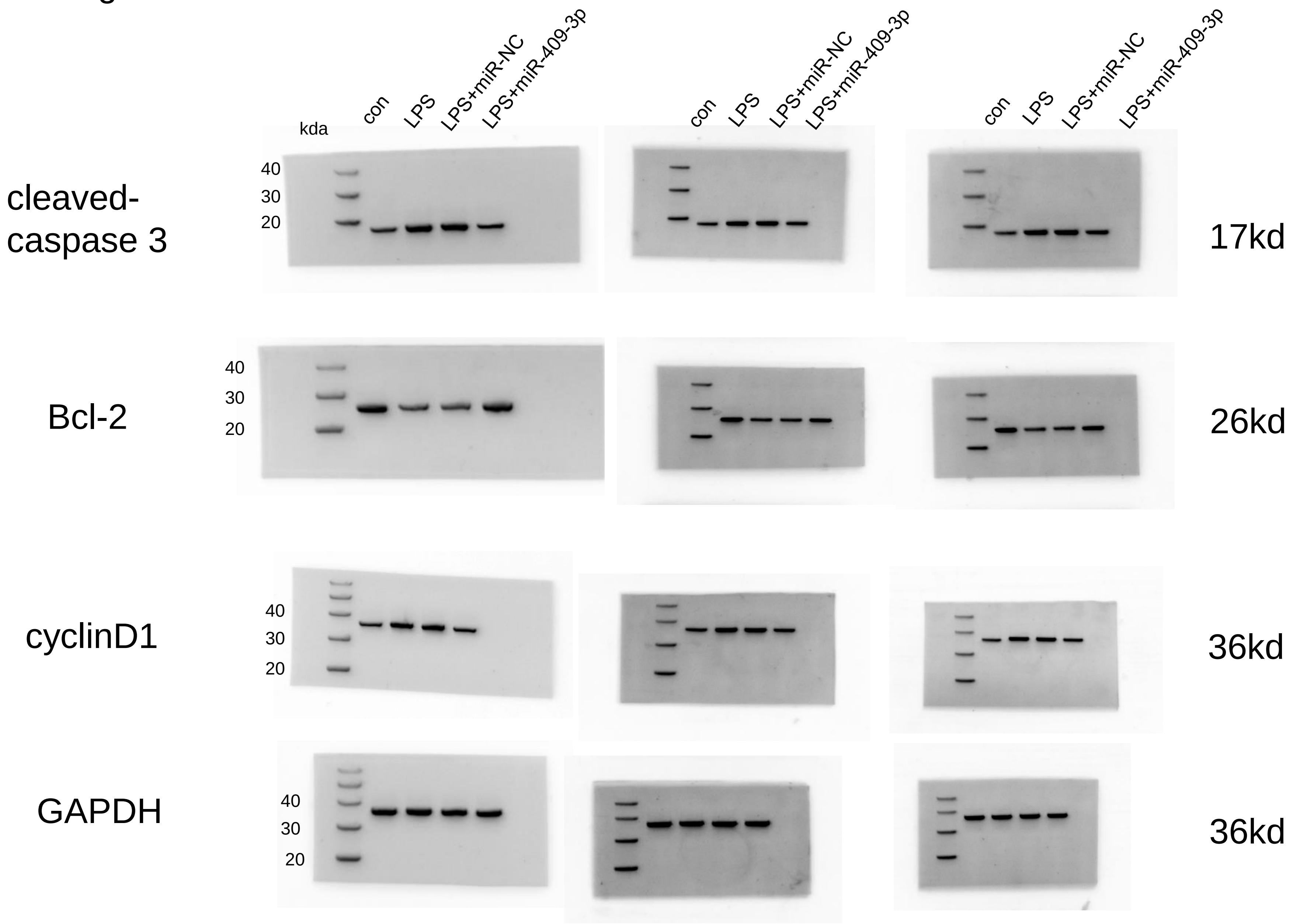
kda
con
LPS
LPS+miR-NC
LPS+miR-409-3p
con
LPS
LPS+miR-NC
LPS+miR-409-3p
con
LPS
LPS+miR-NC
LPS+miR-409-3p
cleaved-caspase 3
40
30
20
17kd
Bcl-2
40
30
20
26kd
cyclinD1
40
30
20
36kd
GAPDH
40
30
20
36kd

Fig 7A

1: LPS+miR-NC   2: LPS+miR-409-3p   3: LPS+miR-409-3p+vector

4: LPS+miR-409-3p+TLR4

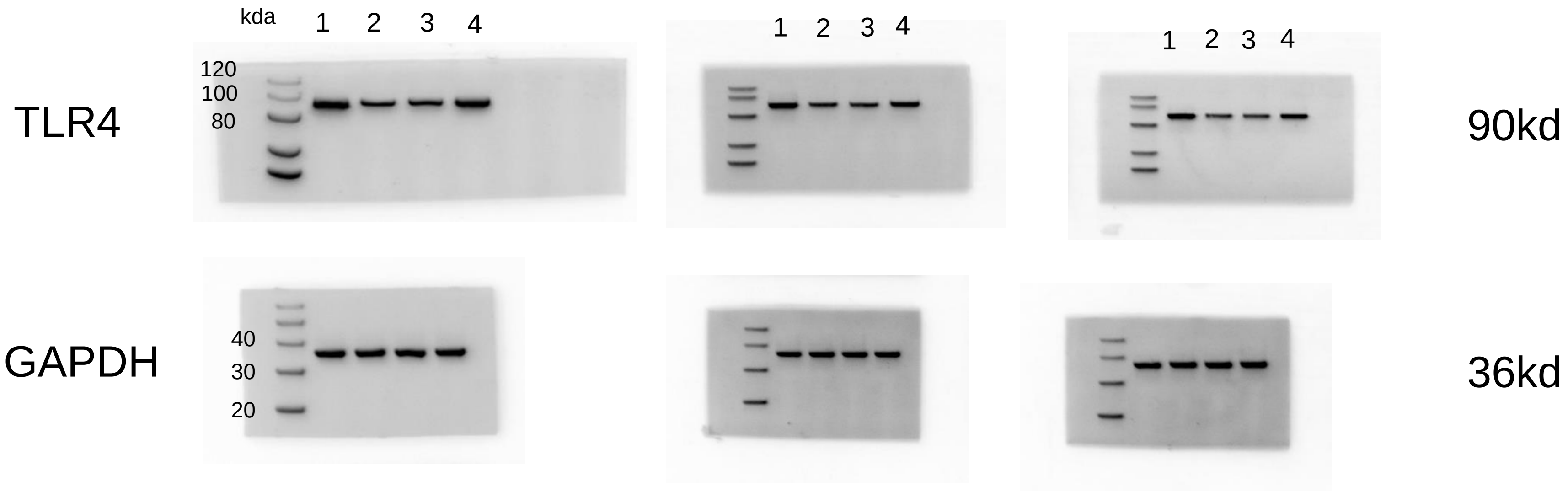

Fig 7F

1: LPS+miR-NC 2: LPS+miR-409-3p 3: LPS+miR-409-3p+vector

4: LPS+miR-409-3p+TLR4

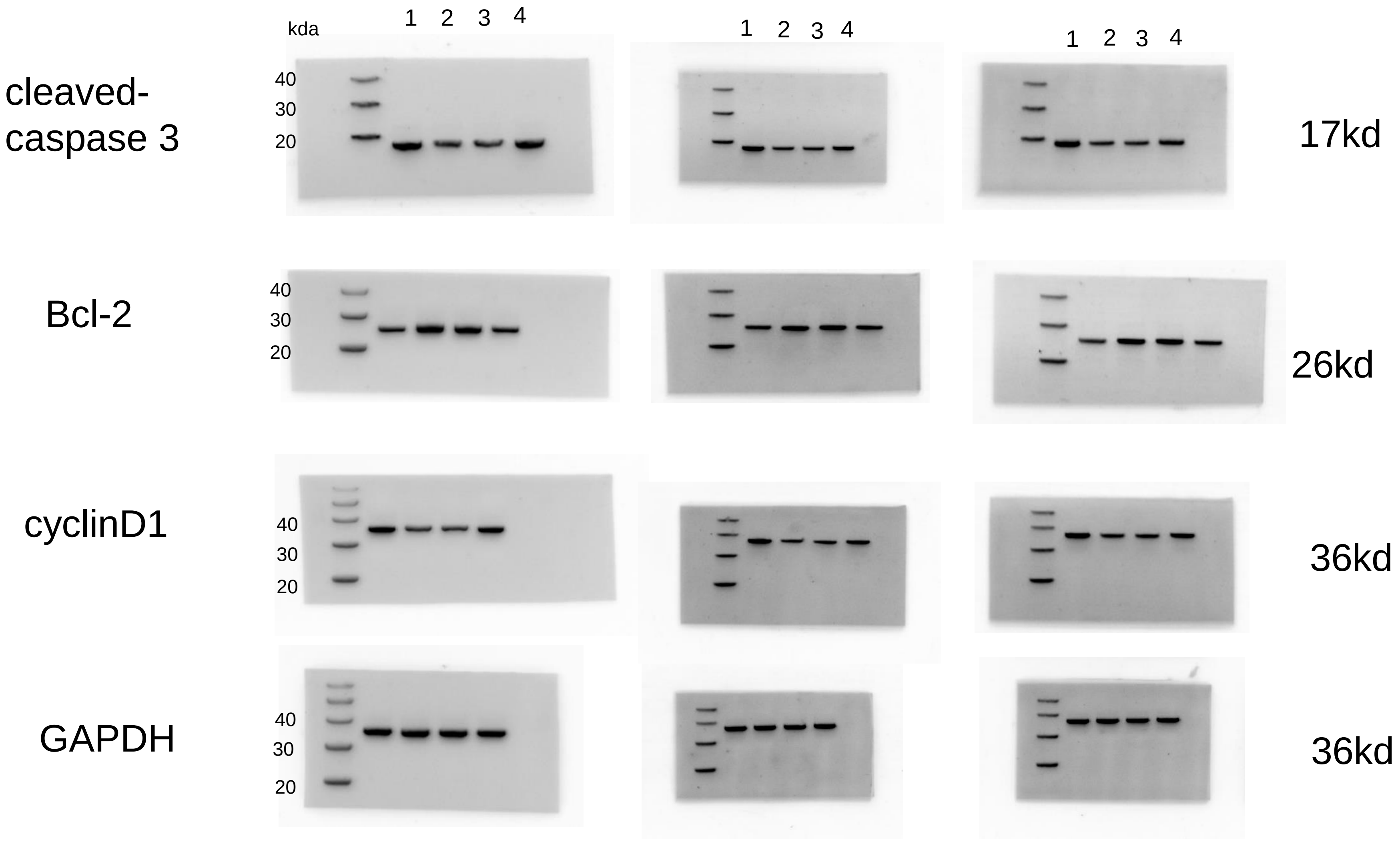

Fig 8A

1: con 2: LPS 3: LPS+si-NC 4: LPS+si-circ_0099630

5: LPS+si-circ_0099630+vector

6: LPS+si-circ_0099630+TLR4

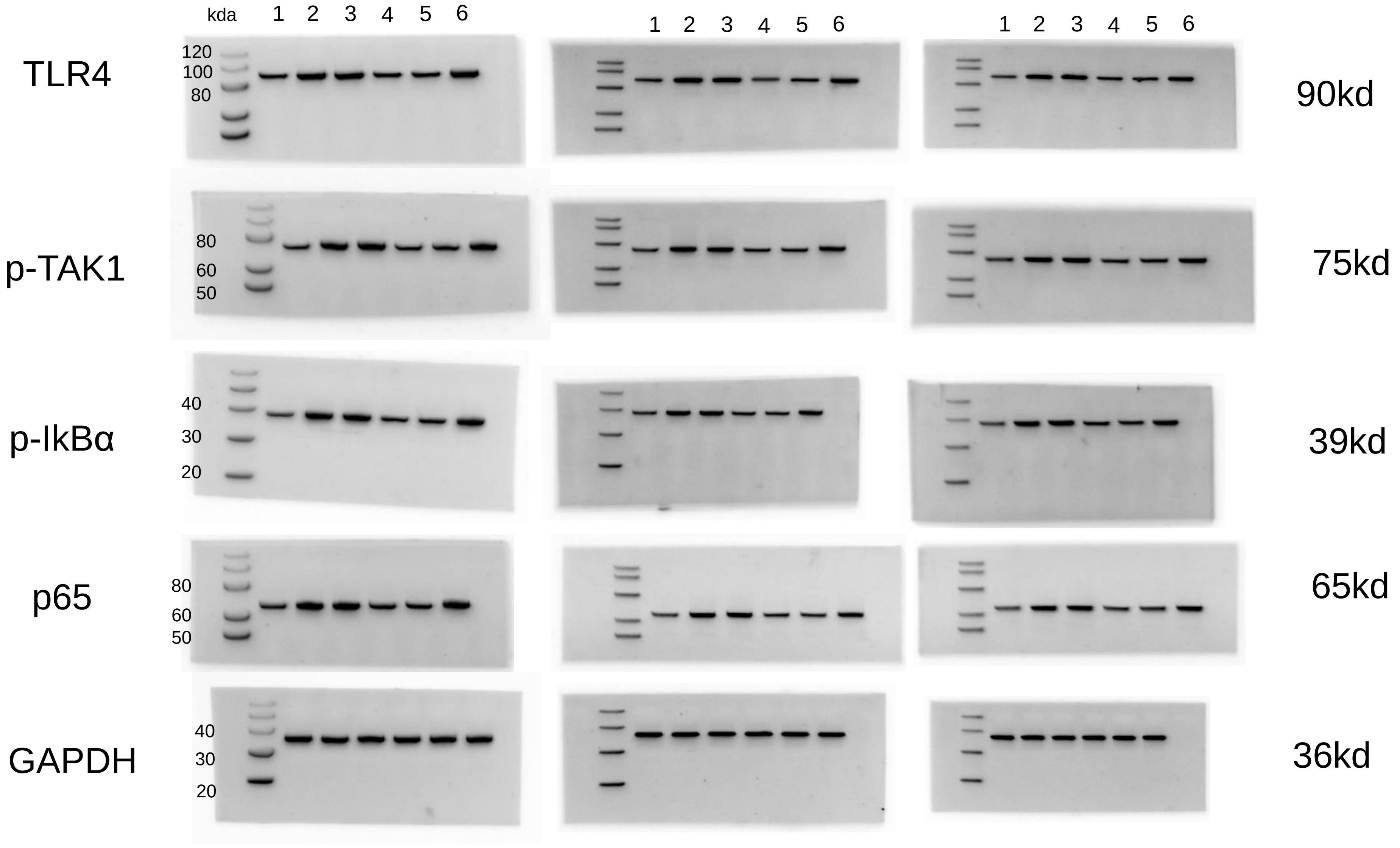

Supplement: Supplementary file 1 — Supplementary Material 1 [file 12903_2023_3622_MOESM1_ESM.pdf]
